# Genome-wide analysis of long non-coding RNAs (lncRNAs) in tea plants (*Camellia sinensis*) lateral roots in response to nitrogen application

**DOI:** 10.3389/fpls.2023.1080427

**Published:** 2023-02-24

**Authors:** Shunkai Hu, Yimeng Hu, Huiling Mei, Jianjie Li, Wei Xuan, Anburaj Jeyaraj, Zhen Zhao, Yuxin Zhao, Rui Han, Xuan Chen, Xinghui Li

**Affiliations:** ^1^ International Institute of Tea Industry Innovation for “One Belt, One Road”, Nanjing Agricultural University, Nanjing, Jiangsu, China; ^2^ College of Resources and Environmental Sciences, Nanjing Agricultural University, Nanjing, Jiangsu, China

**Keywords:** long non-coding RNAs, nitrogen stress, RNA-seq, *Camellia sinensis*, lateral roots

## Abstract

Tea (*Camellia sinensis*) is one of the significant cash crops in China. As a leaf crop, nitrogen supply can not only increase the number of new shoots and leaves but also improve the tenderness of the former. However, a conundrum remains in science, which is the molecular mechanism of nitrogen use efficiency, especially long non-coding RNA (lncRNA). In this study, a total of 16,452 lncRNAs were identified through high-throughput sequencing analysis of lateral roots under nitrogen stress and control conditions, of which 9,451 were differentially expressed lncRNAs (DE-lncRNAs). To figure out the potential function of nitrogen-responsive lncRNAs, co-expression clustering was employed between lncRNAs and coding genes. KEGG enrichment analysis revealed nitrogen-responsive lncRNAs may involve in many biological processes such as plant hormone signal transduction, nitrogen metabolism and protein processing in endoplasmic reticulum. The expression abundance of 12 DE-lncRNAs were further verified by RT-PCR, and their expression trends were consistent with the results of RNA-seq. This study expands the research on lncRNAs in tea plants, provides a novel perspective for the potential regulation of lncRNAs on nitrogen stress, and valuable resources for further improving the nitrogen use efficiency of tea plants.

## Introduction

As a crucial element in plant growth, nitrogen is an essential component of biological compounds such as nucleic acid, protein, chlorophyll, and plant hormones. Nitrogen also regulates plants’ absorption and utilization of other elements, such as phosphorus and potassium (Novoa and Loomis, 1981; Xu et al., 2012). In agricultural production, a large amount of synthetic nitrogen fertilizer has been used in crop fields to promote the yield of crops (Good and Beatty, 2011; Zhang et al., 2012). Nitrogen fertilizer abuse increases agricultural costs and becomes one of the chief culprits of environmental pollution. (Han et al., 2015). Therefore, reducing the application of nitrogen fertilizer and improving the nitrogen use efficiency of crops are momentous to agriculture development. To this end, more and more researchers have carried out studies on crops with low nitrogen tolerance, providing new solutions for the underlying molecular mechanisms of nitrogen regulation and the improvement of nitrogen use efficiency (Kant et al., 2011; Krapp, 2015; Hu et al., 2020). Plant lateral roots (LRs) can absorb nitrogen in the form of organic (amino acids and peptides) and inorganic (nitrate and ammonium nitrogen), and different nitrogen sources can regulate the growth of LRs (Gruffman et al., 2014). The number and location of lateral roots determine the spatial configuration of roots, which means that their formation and development will directly affect plant growth (Duan et al., 2013). Nitrate use was reported to be positively associated with the development of LRs (Lynch, 2013). In other words, reducing the application of nitrate fertilizer can promote the growth of LRs, thereby improving the plant’s nutrient uptake capacity and nitrogen use efficiency (Duan et al., 2013).

Long non-coding RNA (lncRNA) is an RNA transcript longer than 200 nucleotides with no or limited protein-coding capacity (Kung et al., 2013; Kopp and Mendell, 2018). According to their genomic location, they are usually divided into *i* (intron lncRNAs), *o* (overlapping lncRNAs), *u* (intergenic lncRNAs), and *x* (antisense lncRNA) types (Roberts et al., 2011). Much evidence, including epigenetics, transcriptional and post-transcriptional regulation in the form of RNA, can prove that lncRNAs have regulatory functions in gene expression (Sunkar et al., 2007; Caley et al., 2010). Through next-generation sequencing technology and bioinformatics methods, many lncRNAs have been found in *Arabidopsis*, wheat, maize, rice, and other model plants. It is significant in flowering regulation, photomorphogenesis, stress response, and other growth pathways. (Wang et al., 2014; Chekanova, 2015; Wang and Chekanova, 2017; Zhao et al., 2018; Liang et al., 2022). For example, through genome-wide analysis of *Arabidopsis* thaliana full-length cDNA database, 76 ncRNAs were identified, including 5 small interfering RNAs (siRNA) precursors and 14 natural antisense transcripts of protein-coding genes. A set of 127 RNA sequencing samples including total RNAseq datasets and PacBio fl-cDNA datasets in maize was used for identifying 1,077 differentially multiple abiotic stress-responsive TE-lncRNAs, and 39 are hubs in co-expression networks, including a small number that are evolutionary conserved (Lv et al., 2019). In the study of low nitrogen stress in barley, 498 lncRNAs were identified, of which 487 were newly discovered, and 56 lncRNAs responsive to low nitrogen stress were identified (Chen et al., 2020). 637 lncRNAs responsive to nitrogen and 664 lncRNAs responsive to drought were identified in maize seedlings (Zhang et al., 2014; Lv et al., 2016). However, studies of lncRNAs in many non-model plants are relatively limited, and correspondingly, studies on the genome-wide identification and analysis of tea plants lncRNAs are relatively few. Besides, there have been no published studies on the molecular mechanism of lncRNA response to nitrogen in tea plants.

In China, tea (*Camellia sinensis*) is one of the vital cash crops and stands out among traditional industries. As a leaf crop, tea plants consume a lot of nitrogen nutrition and have high requirements for soil nitrogen (Venkatesan et al., 2004; Zhu et al., 2014). The results showed that nitrogen nutrition could promote the germination and elongation of tea shoots, increase the number, weight and area of new shoots and leaves, and advance the tenderness of new shoots (Ruan et al., 2019). Nitrogen can benefit tea plants’ vegetative growth and thwart their reproductive growth, thus increasing yield (Ruan et al., 2019). Within a reasonable dosage range, applying nitrogen in tea gardens can enrich the types of tea aroma substances, improve the freshness of tea leaves, and increase the content of amino acids, tea polyphenols, catechins and chlorophyll, especially free amino acids. (Ruan et al., 2010; Lin et al., 2021).Therefore, improving nitrogen absorption and utilization efficiency has been a focal point of tea nutrition research in recent years. Screening and breeding tea varieties with high nitrogen utilization is paramount to the tea industry (Hu et al., 2020). Most previous studies focused on revealing the coding genes regulated by nitrogen (Xia et al., 2017; Wei et al., 2018). Thanks to the release of the tea genome, we used High-Throughput Sequencing technology to analyze the lncRNAs expression profile of tea roots under high and low nitrogen conditions, analyzed these lncRNAs regulatory coding genes, and identified the function of lncRNAs involved in nitrogen metabolism. In conclusion, our research results provide many valuable references for clarifying the response mechanism of lncRNAs to nitrogen in LRs of tea plants and expand a new path for improving the nitrogen use efficiency of tea plants.

## Materials and methods

### Plant materials and nitrogen treatments

Purebred tea seeds (*Camellia sinensis* cv. Fuding dabai) were germinated in perlite and then cultured in nutrient solution (0.75mM (NH_4_)_2_SO_4_, 0.25 mM Ca(NO3)_2_•4(H_2_O)_3_, 0.05 mM KH_2_PO_4_, 0.35 mM K_2_SO_4_, 0.395 mM CaCl_2_, 0.21 mM MgSO_4_, 35.0 μM NaFeEDTA, 46.1 μM H_3_BO_3_, 2.0 μM MnSO_4_, 0.3 μM CuSO_4_, 2.0 μM ZnSO_4_ and 0.5 μM Na_2_MoSO_4_). Nitrogen was divided into three concentrations: 0.25 mM (labeled as low nitrogen, LN), 1 mM (labelled as control, CK), and 2.5 mM (labelled as high nitrogen, HN). The control experiment (1 mM) was supplemented with 0.75 mM ammonium and 0.25 mM nitrate using (NH4)_2_SO_4_ and Ca (NO3)_2_•4(H_2_O), respectively, which was the best combination of N concentration for seedlings growth. The growth conditions of seedlings in the light culture box were as follows: 28/25°C (day/night), 75% relative humidity, 16/8 h (light/darkness) photoperiod, and 300 μmol^−2^ s^−1^ light intensity. The liquid culture medium was changed every 5 days. The lateral roots of the seedlings were sampled after 10 weeks of seedling growth. The control and nitrogen treatments were repeated three times (CK-1, CK-2, CK-3; LN-1, LN-2, LN-3, HN-1, HN-2, HN-3). The samples were immediately soaked in liquid nitrogen and stored in a refrigerator of -80°C for RNA-seq analysis and qRT-PCR verification.

### RNA isolation, library construction and RNA sequencing

The total RNA of LH, CK, HN was isolated with the plant RNA extraction kit with DNase (TIANDZ, Inc., Beijing, China) according to the manufacturer’s protocol. RNA was purified and concentrated using NanoDrop2000 Spectrophotometer (Thermo Fisher Scientific, USA), 1.2% agarose gel electrophoresis, and Agilent 2100 Bioanalyzer (Agilent Technologies, Inc., Santa Clara, CA, USA). High-quality RNA samples are used for library construction, and then the total RNA-seq library is constructed and sequenced using the IlluminaHiSeq platform. All sequencing data were deposited in the National Center for Biotechnology Information (NCBI) Sequence Read Archive (accession number PRJNA595712).

### Transcriptome assembly

The SolexaQA++ v3.1 program was applied to execute quality trimming using the Q30 value (Cox et al., 2010). After removing rRNA, low-quality reads, aptamer sequences, and contaminating reads, the remaining clean reads were aligned with the reference genome of *C. sinensis* var. *sinensis* by Hierarchical Indexing of Spliced ​​Transcript Alignment (HISat) software (Pertea et al., 2016; Xia et al., 2017). Use the gffCompare program to annotate the assembled transcript and the unknown transcript and then filter out possible lncRNAs by pfam databases (Kong et al., 2007; Han et al., 2016a; El-Gebali et al., 2018).

### Identification of LncRNAs

In this study, a strict calculation method was used to determine the lncRNA of tea plants (Iyer et al., 2015; Xiao et al., 2015). The primary screening of transcripts should meet the following conditions: with a class code of “i”, “x”, “u”, “o”, and “e”, a length ≥ 200 bp, and fragments per kilobase of transcript per million mapped reads (FPKM) value ≥ 0.1(Kelley and Rinn, 2012; Luo et al., 2022). Subsequently, transcripts were compared with uniref90 and Pfam protein databases using the CPC2 program to evaluate their protein-coding potential (Kang et al., 2017). Transcripts that meet these conditions are eventually considered as candidates for lncRNAs for further analysis: Non-coding transcripts larger than 200 bp; FPKM > 1; a CPC score < -1.

### Differential expression analysis

The expression level of lncRNAs was quantitatively detected by FPKM value by StringTie software. DESeq2 software package (1.10.1) was used to analyze the differential expression between nitrogen concentration and control treatments. The P value of the result is adjusted by the methods of Benjamini and Hochberg to control the false discovery rate. The lncRNA with the adjustment value *P* < 0.01 and the absolute value |log2(FPKM) ratio| ≥ 1 by DESeq is considered to be differentially expressed (Wan et al., 2020).

### KEGG pathway enrichment analysis

To elucidate the potential functions of differentially expressed lncRNAs (DE-lncRNAs), co-expression analysis between Nitrogen-responsive lncRNAs and coding genes was employed for generating clusters with different expression patterns. Coding genes of clusters were used for the KEGG pathway enrichment by clusterProfiler (Yu et al., 2012). In the KEGG enrichment analysis, a false discovery rate ≤ 0.05 was used as a criterion to identify significantly enriched pathways (Altschul et al., 1990).

### Validation and quantification of LncRNAs

Total RNA was extracted from the nine samples (CK-1, CK-2, CK-3; LN-1, LN-2, LN-3, HN-1, HN-2, HN-3) using Trizol reagent (Invitrogen). To validate the lncRNAs, Real-time PCR was performed Bio-Rad Real-time thermal cycler CFX96 with SYBR Premix ExTaq™ Kit (Takara Co. Ltd., Japan). The *glyceraldehyde-3-phosphate dehydrogenase* (GAPDH) and *β-actin* were used as controls. The 2^-ΔΔCt^ method was used to determine the relative expression levels (Livak and Schmittgen, 2001). Three biological replicates per sample. All qRT-PCR primers were designed with NCBI primer-BLAST (https://www.ncbi.nlm.nih.gov/tools/primer-blast/). Differences between groups were analyzed by one-way analysis of variance and Duncan’s test, and P < 0.05 was considered to be significantly different between treatment groups.

## Result

### Phenotypic response of *C.sinensis* lateral roots to nitrogen treatments

**Figure 1:**
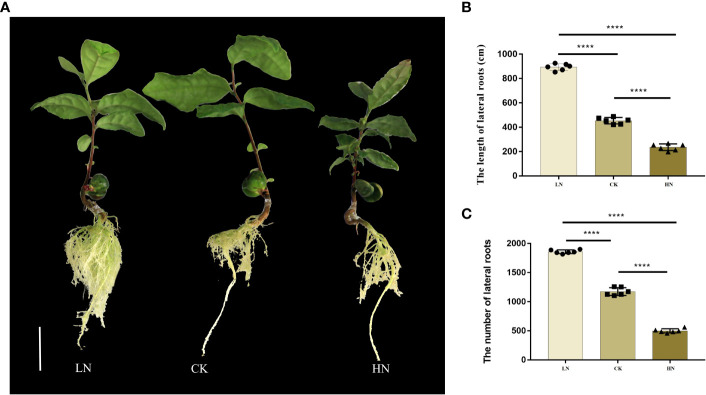
**(A)** Lateral roots (LRS) phenotype of low-nitrogen (LN), normal-nitrogen (CK) and high-nitrogen (HN). **(B)** The length of LRs in hydroponics culture. **(C)** The number of LRs in hydroponics culture. Scale bars = 2 cm. Values are mean ± S.D. *P ≤ 0.5, **P ≤ 0.01, ***P ≤ 0.001, ****P ≤ 0.0001, (Student’s t-test).

In different nitrogen treatments, the growth of the underground part of the seedlings changed significantly in the tenth week (**Figure 1A**). The main results were as follows: with the increase in nitrogen concentration, the number and the length of lateral roots decreased significantly (**Figure 1B**, **C**). This showed that low nitrogen conditions could promote the growth of lateral roots of seedlings. On the contrary, it was inhibited under high nitrogen conditions.

### Genome−wide prediction of LncRNAs candidates in *C*. *sinensis*

**Figure 2:**
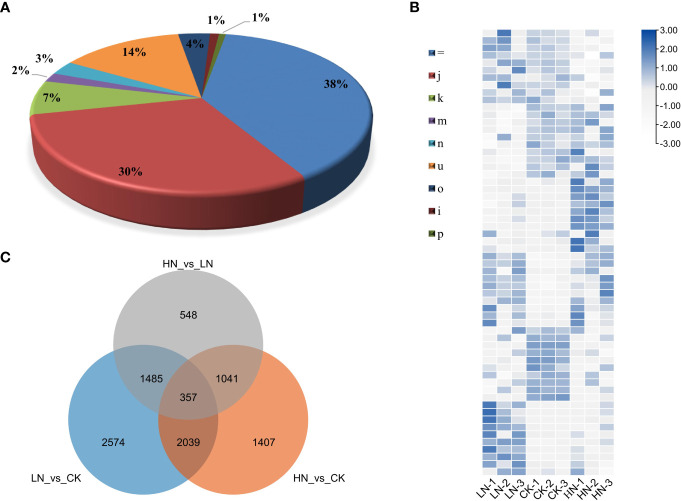
**(A)** Compare the category code generated by CuffCompare with the tea plants genome, and then calculate the percentage. “=“: complete, exact match of intron chain; “j”: multi-exon with at least one junction match; “k”: containment of reference (reverse containment); “m”: retained intron (s), all introns matched or retained; “n”: retained introns (s), not all introns matched/covered.; “u”: none of the above (unknown, intergene); “o”: other same strand overlap with reference exons; “i”: fully contained within a reference intron; “p”: possible polymerase run-on (no actual overlap). (b~c) Analysis of transcripts in LRs (lateral roots) of tea plant under three nitrogen treatments, LN, CK and HN, with three replicates for each. **(B)** Cluster heat map of DE-lncRNAs (differentially expressed lncRNAs) in three treatments. **(C)** Venn diagram of common DE-lncRNAs.

To investigate the lncRNAs in tea plants under nitrogen treatments, transcriptome sequencing analysis of three biological repeats of HN, CK and HN was carried out using the Illumina HiSeq2500 platform. Each sample had an average base of 9GB, and the lowest value of Q30 was 92.83% (**Table S1**). All clean reads were compared with the tea plants reference genome. A total of 86814 transcripts were identified, and the results showed that 38% (33515) of transcripts matched exactly and entirely with the intron chain and 30% (26279) were annotated as multi-exon with at least one junction match, while 7% (6049) transcripts were annotated as containment of reference (reverse containment) (**Figure 2A**). Total RNA-seq technology could recover transcripts located in gene intergenic or intronic regions. After filtering the data through CPC2 and Pfam, a total of 16,452 candidate transcripts were obtained.

### DE-lncRNAs under nitrogen treatments

**Figure 3:**
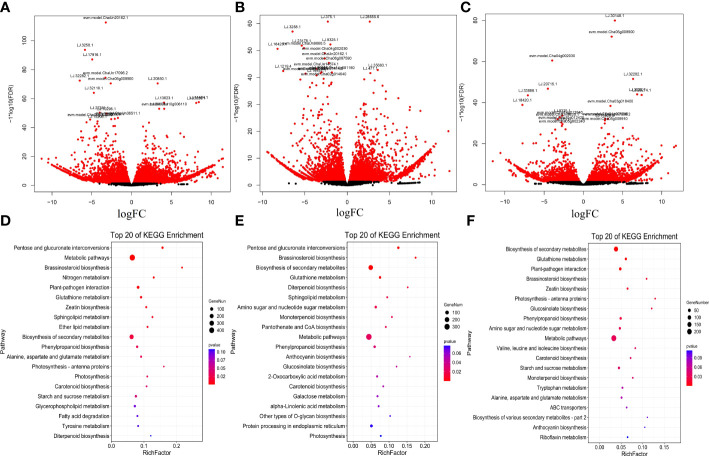
**(A-C)** Volcano map of DE-coding genes in LRs of tea plants under different nitrogen conditions. **(A)** LN vs CK. **(B)** HN vs CK. **(C)** HN vs LN. (d~f) KEGG pathways analysis. Top 20 pathways for the predicted coding genes of DE-coding genes. **(D)** LN vs CK. **(E)** HN vs CK. **(F)** HN vs LN.

To identify nitrogen-responsive lncRNAs in LRs of tea plants, the expression of lncRNAs was compared between the LN, CK and HN treatments. Based on the standardized FPKM value, 9,451 DE-lncRNAs were revealed in LN, CK, and HN treatments (**Figure 2B**). Moreover, 357 DE-lncRNAs overlapped in those treatments (**Figure 2C**). Furthermore, in the comparison of LN and CK treatments, there were 6,455 DE-lncRNAs, of which 3,805 were up-regulated and 2,650 were down-regulated (**Figure 3A**); in the comparison of HN and CK treatments, there were 4,844 DE-lncRNAs, of which 2,879 were up-regulated and 1,965 were down-regulated (**Figure 3B**); and 3,431 DE-lncRNAs were identified in the comparison of HN and LN, of which 1,780 were up-regulated and 1,651 were down-regulated (**Figure 3C**).

### Enrichment analysis of nitrogen-responsive coding genes

The nitrogen-responsive coding genes were submitted into KEGG pathway enrichment, and the top 20 enriched pathways were shown in (**Figure 3D-F**). In the LN vs. CK comparisons, 2,161 coding genes were annotated in 128 KEGG pathways. Among them, the most enriched pathway was metabolic pathways (447), followed by biosynthesis of secondary metabolites (248), photosynthesis-antenna proteins (61) and starch and sucrose metabolism (35) (**Figure 3D**, **Table S2**). In addition, 1,514 coding genes were enriched in 116 KEGG pathways between HN vs. CK comparisons. Coding genes identified in metabolic pathways (327) were the most abundant, followed by biosynthesis of secondary metabolites (197), protein processing in endoplasmic reticulum (42) and amino sugar and nucleotide sugar metabolism (28) (**Figure 3E**, **Table S3**). Among the LN vs. HN comparisons, 1,140 coding genes were verified in 113 KEGG pathways. Moreover, the most significant pathway was metabolic pathways (242), followed by Biosynthesis of secondary metabolites (151), plant-pathogen interaction (36) and glutathione metabolism (20) (**Figure 3F**, **Table S4**).

### Co-expression clustering analysis of nitrogen-responsive LncRNAs and coding genes in lateral roots development

**Figure 4:**
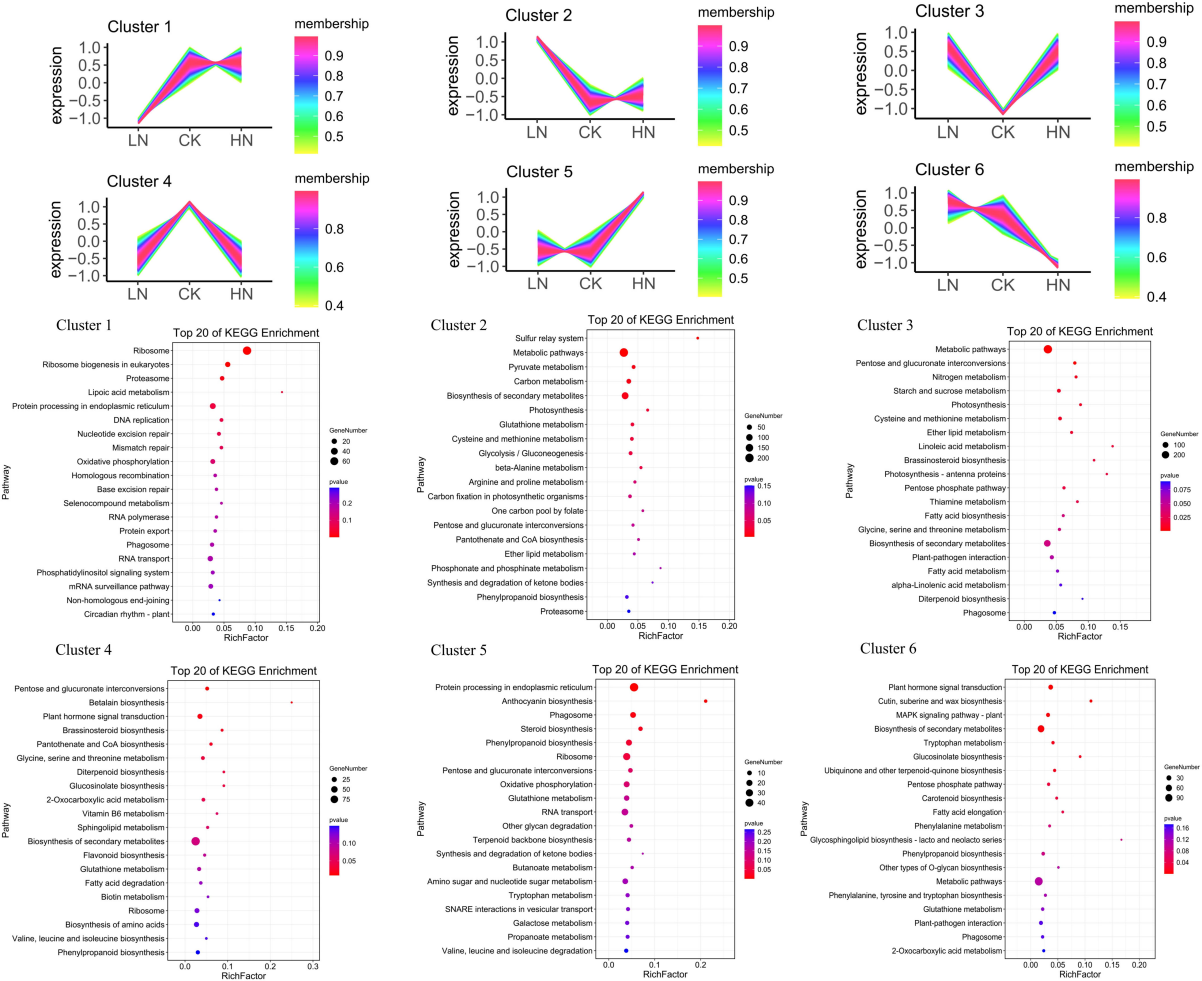
Nitrogen-responsive lncRNAs enrichment plot from KEGG pathway analysis for different coexpressed clusters.

In different nitrogen treatments, the lncRNAs with the same trend of coding genes expression were selected and annotated by KEGG (**Figure 4**). In LN treatment, compared with CK and HN treatments, the up-regulated and down-regulated DE-lncRNAs were clustered into cluster1 and cluster2, respectively, and then these up-regulated and down-regulated DE-lncRNAs were annotated to cluster 1 and cluster 2 KEGG pathways, severally. The down-regulated DE-lncRNAs compared with CK and HN was shown in cluster1. The cluster 1 KEGG pathway identified that 720 DE-lncRNAs were annotated in 104 KEGG pathways. The pathway with the most aggregation was ribosome (65), followed by protein processing in endoplasmic reticulum (26) and RNA transport (20) (**Table S5**); the up-regulated DE-lncRNAs were shown in cluster 2, and KEGG pathway (cluster 2) identified that 1311 DE-lncRNAs were annotated in 122 KEGG pathways, such as metabolic pathways (275), biosynthesis of secondary metabolites (145) and plant-pathogen interaction (32) (**Table S6**). In CK treatment, compared with LN and HN treatments, the up-regulated and down-regulated DE-lncRNAs were clustered into cluster 3 and 4, respectively, and then these up and down-regulated DE-lncRNAs were annotated to cluster 3 and cluster 4 KEGG pathways, severally. The down-regulated DE-lncRNAs were shown in cluster 3. The KEGG pathway (cluster 3) identified that 971 DE-lncRNAs were annotated in 121 KEGG pathways, such as metabolic pathways (202), biosynthesis of secondary metabolites (114) and carbon metabolism (38) (**Table S7**); the up-regulated DE-lncRNAs were shown in cluster 4, and KEGG pathway (cluster 4) identified that 815 DE-lncRNAs were annotated in 116 KEGG pathways, such as biosynthesis of secondary metabolites (99), biosynthesis of amino acids (25), plant hormone signal transduction (24) (**Table S8**). In HN treatment, compared with LN and CK treatments, the up-regulated and down-regulated DE-lncRNAs were clustered into cluster 5 and cluster 6, respectively, and then these up and down-regulated DE-lncRNAs were annotated to cluster 5 and cluster 6 KEGG pathways, severally. The up-regulated DE-lncRNAs were shown in cluster 5, and KEGG pathway (cluster 5) identified that 993 DE-lncRNAs were annotated in 114 KEGG pathways, such as protein processing in endoplasmic reticulum (45), ribosome (30) and RNA transport (25) (**Table S9**); the down-regulated DE-lncRNAs were shown in cluster 6, and KEGG pathway (cluster 6) identified that 556 DE-lncRNAs were annotated in 102 KEGG pathways, such as metabolic pathways (114), biosynthesis of secondary metabolites (74), and plant hormone signal transduction (25) (**Table S10**). The clustering results showed that nitrogen treatment could affect the expression of lncRNAs, associated with the nitrate transporter and controlling the growth of tea plants LRs. In addition, nitrogen treatment regulated the hormone signals of tea plants LRs, associated with auxin synthesis and auxin signals genes.

### Validation of lncRNAs expression using qRT-PCR

**Figure 5:**
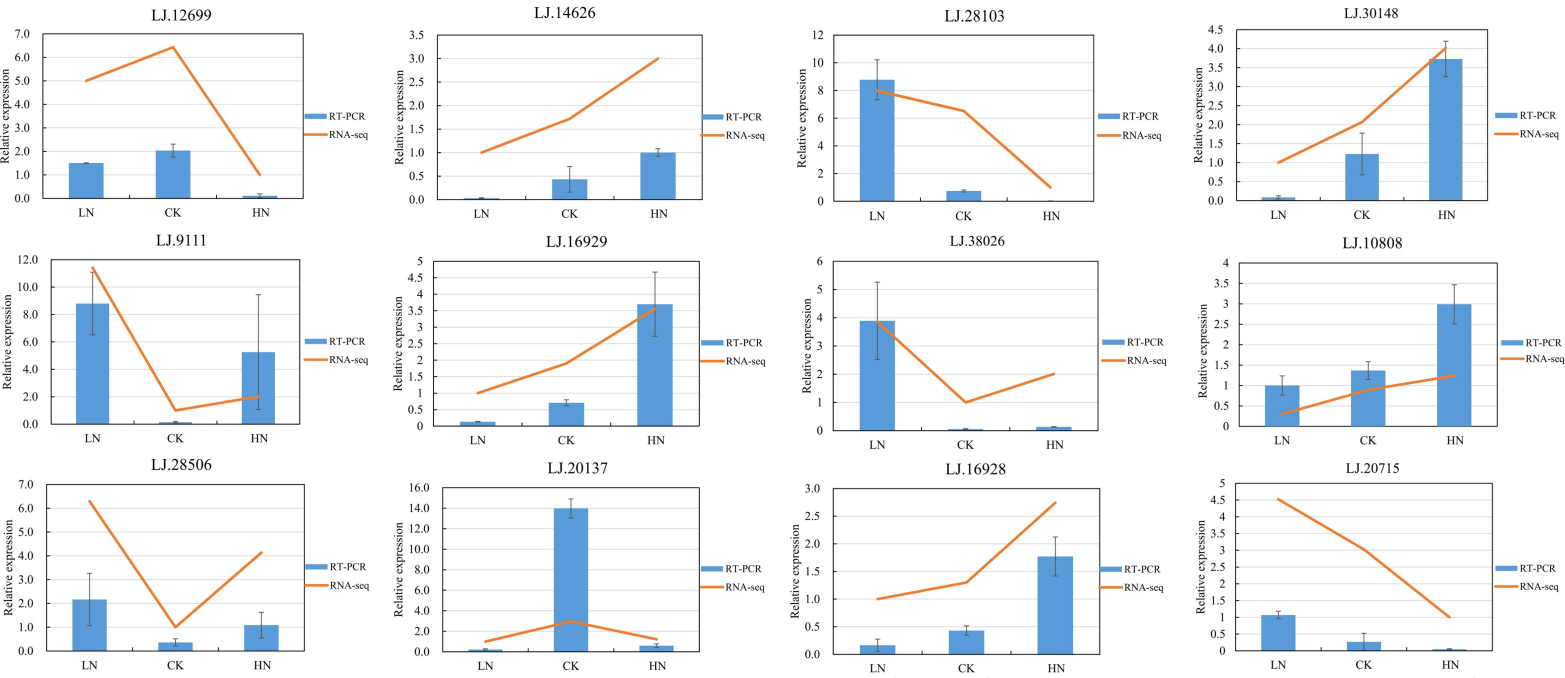
Comparison of expression profiles of selected lncRNAs as determined by Real Time-PCR.

To determine the reliability of RNA-seq transcriptome results, 12 DE-lncRNAs were selected for qRT-PCR verification. The result showed that the expression level of these DE-lncRNAs was in line with that of transcripts estimated from sequence data, suggesting the repeatability and accuracy of RNA-seq data (**Figure 5**). The identified DE-lncRNAs sequences were recorded in **Table S11**.

## Discussion

Improving nitrogen use efficiency is of great significance in agricultural production and environmental protection.To promote nitrogen use efficiency and adapt to barren land, improving the resistance mechanism to low nitrogen or nitrogen deficiency is advisable. (Han et al., 2015). Previous studies have shown that the early nodulin gene was found in rice, which was identified by transcriptional analysis under low nitrogen stress and was related to increased nitrogen use efficiency (Bi et al., 2009). At the same time, barley is becoming a model crop for similar research because of its adaptability to barren land (Chen et al., 2018). Due to the particularity system of tea plants, nitrogen use efficiency is critical in recent research (Zhang et al., 2020). It includes nitrogen absorption efficiency, utilization efficiency and transport efficiency.

During past years, research on improving the nitrogen use efficiency of tea plants has been the top priority. In addition, nitrogen can also regulate the synthesis and transport of auxin, gibberellin, and other plant hormones through nitrogen signals, thus affecting the growth and development of plant LRs (Sun et al., 2017). In previous studies, nitrogen deficiency induced the expression of high-affinity nitrate transporters NRT1, NRT2.4 and NRT2.5 in *Arabidopsis thaliana* roots (Lezhneva et al., 2014). Moreover, NRT1 and NRT2 can transport not only ${\text{NO}}_{3}^{-}$ but also auxin. So, under a low nitrogen environment, NRT1 and NRT2 are highly expressed and regulate the formation of LRs by inducing auxin accumulation and transport (Krouk et al., 2010). In this study, low nitrogen could also promote the growth of tea plants’ LRs. However, accumulating evidence shows that lncRNAs are momentous in various plants’ biological processes, but their characteristics, expression patterns and potential functions in response to nitrogen in tea plants have not been reported. In this study, we identified the lncRNAs of tea plants according to the latest genome of tea plants, and confirmed the lncRNAs related to nitrogen response and LRs development. This study has gained discoveries of the regulatory function of lncRNAs on nitrogen in tea plants and provides a resource for further study of the molecular mechanism of nitrogen response.

In previous studies, 7,245 lncRNA were identified in RNA-seq analysis under nitrogen-deficiency treatment in maize, of which 673 were corresponding to low nitrogen deficiency (Lv et al., 2016). 918 differentially expressed lncRNAs was found in the study of nitrogen deficiency in rice (Shin et al., 2018). In the research on nitrogen deficiency in *Populus*, 126 differentially expressed lncRNAs were identified in N-starved (Chen et al., 2016). In this study, 16452 candidates lncRNAs revealed in LN and CK and HN treatments were selected from 9 LRs samples of tea plants. The results showed that 9451 lncRNAs with different functions were differentially expressed under nitrogen treatments. Therefore, these lncRNAs are involved in the response of tea plants to nitrogen stress, and the results can provide candidate genes for the study of nitrogen utilization in tea plants.

In this study, the results of KEGG pathway analysis showed that the DE-lncRNAs of the three treatments existed in multiple KEGG pathways, and the top three were “metabolic pathways”, “biosynthesis of secondary metabolites”, and “glutathione metabolism” (**Figure 4**). The results not only revealed that these pathways were related to nitrogen stress response, but also indicated that some components were involved in nitrogen stress response processes simultaneously. In addition, each group has specific DEGS and enrichment pathways, which means that there are different response mechanisms under different N stress. In addition, the up-regulated and down-regulated DE-lncRNAs of each group compared with the other groups were analyzed by KEGG pathway. The results demonstrated response mechanism varied with different N stresses.

In LN treatment, compared to the other two groups (CK, HN), up-regulated DE-lncRNAs were mainly annotated in “metabolic pathways” and “biosynthesis of secondary metabolites”. This indicated that nitrogen deficiency could promote the expression of coding genes in these two pathways. Considering the involvement of stress response: It has been reported that for many plant species, an increase in plant secondary metabolism caused by biotic or abiotic stress is part of the stress response (Sathiyabama et al., 2016). In line with this, previous studies manifested that the production of terpenoids and cannabinoids in plants is stimulated under the stress of nitrogen and phosphorus nutrient deficiency. The results showed a negative correlation between secondary metabolism and plant stress. That is, the concentration of secondary metabolites decreased with the increase of nitrogen supply, and the optimal nitrogen condition and high nitrogen condition in plants have nothing to do with secondary metabolism (Shiponi and Bernstein, 2021a; Shiponi and Bernstein, 2021b). The result is supported by many other plant studies. Furthermore, the carbon nutrient balance hypothesis points out that the production of nitrogen-rich primary metabolites is limited under low nitrogen conditions. Plant metabolism and energy consumption changed from the production of nitrogen-containing metabolites to the production of nitrogen-free metabolites, promoting the accumulation of secondary metabolites (Massad et al., 2012; Albornoz, 2016). This result is also consistent with the results of DE-lncRNAs analysis in our study.

Low-affinity transport system (LATS) and high-affinity transport system (HATS) are two NO3 uptake systems in plants They are mediated by nitrate transporter 1 (NRT1) and nitrate transporter 2 (NRT2), respectively (Tong et al., 2005; Wang et al., 2012). The results showed that with the decrease in nitrogen use efficiency, high-affinity nitrate transporters (NRT2) activity replaced low-affinity nitrate transporters (NRT1) activity, so NRT2 may be the key target to improve nitrogen use efficiency, especially under the condition of low nitrogen condition (Huang et al., 1999). Moreover, studies have shown that under low nitrogen conditions, NRT2 not only transports NO_3_, but also participates in the transport of auxin, thus promoting plant root growth (Krouk et al., 2010). In this study, the up-regulated DE-lncRNA in low nitrogen treatment were clustered in the “nitrogen metabolism” pathway, including LJ.29731 and LJ.29732 corresponding to coding genes NRT2.3 and NRT2.4. This indicates that lncRNAs cis-regulate the expression of corresponding NRT2 coding genes under low nitrogen treatment and participate in the growth of lateral roots of tea plants (**Figure 1**, **Figure 4**, cluster2).

Plant hormones can regulate plant growth through different signal pathways and contribute to cell elongation, seed germination, flower formation, leaf morphogenesis, fruit development and so on. The same is true for resistance to biotic and abiotic stress (Qaadt, 2020). In this study, the normal nitrogen (CK), compared with the other two groups (LN and HN), up-regulated DE-lncRNAs were clustered in the “plant hormone signal transduction pathway”. Auxin is a crucial signal of plant growth and the higher the concentration, the more inhibited it is. At the same time, the precise distribution of auxin in plants determines the top-down organ morphology of plants (De Smet et al., 2010). AUX/IAA family transcription factors and auxin response factor (ARF) are two main transcription factors in the signal transduction pathway. In the auxin signal transduction pathway, ARF binds to the auxin response element in the auxin response gene promoter to activate the expression of the auxin response genes (Figueiredo and Strader, 2022). At low auxin concentration, AUX/IAA binds to ARF to form a heterodimer, which inhibits the transcription of ARFs, and then the expression of ARF-regulated auxin-responsive genes was inhibited. When auxin rises to a certain concentration, auxin binds to its receptor SCF-TIRl (Han et al., 2020). By interacting with AUX/IAA, AUX/IAA proteins were ubiquitinated and ARFs were released. Thus, auxin response genes are expressed and plants undergo a series of growth responses (Figueiredo and Strader, 2022). In this study, compared with CK and HN treatments, 14 DE-lncRNAs coding to ARF genes were up-regulated in LN treatment, which indicated that lncRNAs regulated the expression of ARF genes in low nitrogen treatment, thus increasing the transcriptional activity of ARFs (**Table S12**). Jasmonic acid (JA) and their derivatives methyl jasmonate (MeJA) play an essential role in plant morphological, physiological and biochemical processes in response to drought, cold and salt stress (Ali and Baek, 2020; Zhang et al., 2021). Different nitrogen concentrations are also involved in the metabolism and synthesis of jasmonic acid. In previous studies, JAR1 (jasmonate resistant 1)-activated JA and ethylene signaling pathways could inhibit roots growth in Boron deficiency, and low nitrogen could inhibit the content of jasmonic acid (Li et al., 2020; Huang et al., 2021). In this study, three DE-lncRNAs (LJ.13289, LJ.13289, LJ.33982) corresponding to jasmonate resistant 4 were up-regulated in LN treatment. SAURs were discovered and named because of their response to the rapid induction of auxin. The expression of SAURs was also regulated by many internal and external factors (Wiesel et al., 2015). In addition to being induced by auxin, a small part of the expression of SAURs showed auxin inhibition or non-response (Ren and Gray, 2015). Many studies have shown that SAURs can also be induced by other plant hormones, such as brassinolide, gibberellin and cytokinin, while abscisic acid and jasmonic acid inhibit it *(* Bai et al., 2012). In addition, light, temperature, moisture and other environmental factors also affect the expression of SAURs (Ouyang et al., 2015). In this study, 7 DE-lncRNAs coding to SAUR genes were down-regulated in HN treatment compared with LN and HN treatments (LJ.1615, LJ.12746, LJ.11860, LJ.5020, LJ.12505, LJ.23544, LJ.36187). To sum up, DE-lncRNAs are involved in the synthesis and metabolism of hormones in tea plants under different nitrogen conditions.

Glutathione is a reductant that regulates signal molecules and scavenges free radicals and ROS in the redox system of plant cells. Glutathione participates in the metabolic process of all eukaryotes to improve plants’ tolerance to abiotic stresses such as salt, temperature, drought, heavy metals and autotoxicity (Hodges et al., 1996; Koprivova et al., 2010; Niu et al., 2017). Glutathione adjusts continuously in plants, giving play to different metabolic processes and extremely significant antioxidant advantages, which can effectively scavenge free radicals in organisms, which cannot be reflected by glutamine, lycopene and other oxidants (Nakamura et al., 2019). Glutathione can oxidize GSH by reactive oxygen species and regulate the reduction of GSSG by glutathione reductase, thus regulating the balance between GSH (reduced) and GSSG (oxidized) (Bashandy et al., 2010). Exogenous GSSG could not induce root growth under normal conditions but could promote root development under auxin treatment (Tyburski and Tretyn, 2010). Therefore, the interaction between auxin and GSSG can regulate plant root development. It is reported that plants can inhibit the growth of lateral roots by regulating the reduction ratio of GSH/GSSG in the presence of auxin (Wei et al., 2013). Glutathione S-transferase (GSTs) converts GSH to GSSG, while glutathione reductase (GR) induces the reduction of GSSG to GSH, so glutathione S-transferase plays an essential role in plant growth and development (Jiang et al., 2010). In this study, cluster analysis of different treatments showed that a total of 82 De-lncRNAs coding glutathione S-transferase genes were distributed in six clusters, indicating that in nitrogen treatment, lncRNAs could regulate the coding glutathione S-transferase genes and participated in the growth and development of tea plants (**Figure 4**, **Table S13**).

## Conclusion

Through RNA-seq and bioinformatics, this study identified 16,452 LncRNAs in the whole genome of tea plants, among which 9,451 DE-lncRNAs were differentially expressed under nitrogen stress. The KEGG pathway further analyzed the biological and regulation function of these lncRNAs. To sum up, this study expands the cognition of lncRNAs in tea plants and explores a path for further study of tea plants response to nitrogen and improvement of nitrogen use efficiency of woody plants.

## Data availability statement

The data presented in the study are deposited in the National Center for Biotechnology Information (NCBI) Sequence Read Archive repository, accession number PRJNA595712.

## Author contributions

SH: Project administration. Methodology, Formal analysis, Investigation, Data curation, Writing-original draft. XL: Methodology, Formal analysis, Investigation, Data curation, Writing-original draft. XC: Formal analysis, Investigation. WX: Formal analysis, Data curation, Writing-original draft. YH: Investigation, Visualization. JL: Investigation. HM: Conceptualization. ZZ: Formal analysis. YZ: Investigation. AJ: Visualization. RH: Formal analysis. All authors contributed to the article and approved the submitted version.
