# Supplementary material for: Genome-wide analysis of long non-coding RNAs (lncRNAs) in tea plants (Camellia sinensis) lateral roots in response to nitrogen application
Source: Front Plant Sci. 2023 Feb 24;14:1080427. doi: 10.3389/fpls.2023.1080427 (PMC9998519; doi:10.3389/fpls.2023.1080427)
Supplement: Supplementary file 1 [file Table_1.docx]

Table 1 | Overview of the genome alignment result.

| **Samples** | **Read Number** | **Base Number** | **%≥Q30** |
| --- | --- | --- | --- |
| LN-1 | 22300039 | 6690011700 | 93.81 |
| LN-2 | 22352271 | 6705681300 | 94.08 |
| LN-3 | 71345574 | 21403672200 | 92.83 |
| CK-1 | 50566454 | 15169936200 | 93.86 |
| CK-2 | 26485890 | 7945767000 | 94.03 |
| CK-3 | 51958794 | 15587638200 | 94.57 |
| SN-1 | 22116811 | 6635043300 | 93.97 |
| SN-2 | 23245627 | 6973688100 | 93.64 |
| SN-3 | 22586710 | 6776013000 | 93.67 |
